# Intracardiac thrombus formation and thromboembolic events in children with cardiomyopathies: A single‐center case series

**DOI:** 10.1002/ccr3.2972

**Published:** 2020-05-31

**Authors:** Abdulrahman Bendahmash, Saeed Almanie, Abdullah Alwadai

**Affiliations:** ^1^ Department of pediatric cardiology, Heart Center King Faisal Specialist Hospital and Research Centre Riyadh Saudi Arabia

**Keywords:** cardiomyopathy, children, intracardiac thrombosis, thromboembolism

## Abstract

Intracardiac thrombosis and distant thromboembolic events are rare complications of patients diagnosed with any type of cardiomyopathies. The low prevalence of this entity makes it challenging and unfortunate for the patients and their families. This review aims to add to the current limited available data describing similar clinical entities.

## Graphical abstract

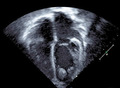

## 1 INTRODUCTION

Cardiomyopathy is a disorder of the heart muscle carrying a great potential of resulting in major cardiac complications and overall health deterioration. It is considered as the most common cause of heart failure and heart transplantation in infants and older children.^1^

The American Heart Association (AHA) had issued a project to precisely define heart muscle diseases. Since cardiomyopathy is frequently encountered in pediatric cardiology practice, it is important to accurately define this condition. The expert consensus panel defined cardiomyopathies as “heterogeneous group of diseases of the myocardium associated with mechanical and/or electrical dysfunction that usually (but not invariably) exhibit inappropriate ventricular hypertrophy or dilatation and are due to a variety of causes that frequently are genetic. Cardiomyopathies either are confined to the heart or are part of generalized systemic disorders, often leading to cardiovascular death or progressive heart failure‐related disability".^2^

The epidemiology of cardiomyopathy held the interest of many researchers since not enough data was available in the literature before the nineties.^1^, ^3^ In one of the most extensive studies that investigated the incidence of pediatric cardiomyopathy in the United States, the annual incidence was found to be 1.13 cases per 100 000 children.^1^

Although the vast majority of cardiomyopathy cases are referred to as idiopathic, certain causes like genetic disorders, post myocarditis, and inborn errors of metabolism are identified in one‐third of pediatric patients presenting with cardiomyopathies.^3^

Despite the great effort made by the researchers in recent years, the complications related to cardiomyopathy have not been fully investigated in pediatric patients where the focus was almost always on the prognosis of the disease rather than the short‐term complications. Generally, the outcome of a patient with cardiomyopathy depends mainly on the underlying cause and the course of the disease.^2^

McCrindle et al from the University of Toronto, the hospital for sick children in Canada published a study in 2006 about cardiac thrombosis in children with cardiomyopathy which involved 66 patients presenting with cardiomyopathy out of which four patients were found to have a cardiac thrombus at presentation and another four subjects developed a cardiac thrombus during subsequent follow‐up in no later than 18 months from the first presentation. The authors concluded that the risk of thrombosis in children with dilated cardiomyopathy is significant and unpredictable as thrombus formation might occur despite the use of anticoagulation.^4^, ^5^

Department of pediatric cardiology in Gaziantep University, Turkey, has conducted a retrospective review about intracardiac thrombus in children with cardiomyopathy published by Ahmet İrdem et al The paper had illustrated that out of 83 patients with dilated cardiomyopathy who were followed between June 2004 and December 2011, five patients were found to have an intracardiac thrombus. The study was also questioning the relation between left ventricular ejection fraction (LVEF) and intracardiac thrombus formation where the patients were divided based on the LVEF (either > 30% or < 30%). Surprisingly, there was no statistical significance between the groups (*p* = .910) so the study concluded that initiation of anticoagulants is recommended regardless of the LVEF.^6^

This article aims to describe the nine cases of intracardiac thrombi or cardiac‐related thromboembolic events in patients who are known to have cardiomyopathies following up in our institution. We would like to contribute by adding to the limited worldwide and regional data about this fatal complication. In our study we will identify all pediatric patients with cardiomyopathy who had either an intracardiac thrombus or distant embolus and describe their course of the disease, trying to find risk factors for the development of the thrombus, then we will highlight the management and the outcomes.

## 2 METHODOLOGY

This is a retrospective cohort study, chart review for all pediatric patients who were diagnosed with cardiomyopathy and developed an intracardiac thrombus, or had a thromboembolic event using echocardiography as a tool for diagnosis in King Faisal Specialist Hospital and Research Centre (KFSHRC), Riyadh, Saudi Arabia. The definition and diagnostic criteria of cardiomyopathy were based on the American Heart Association (AHA) scientific statement regarding the classification and diagnosis of cardiomyopathy in children based on the morpho‐functional approach with a structural and functional phenotype.^7^

Inclusion criteria:

- All pediatric patients (from 1 day to 14 years of age) who are diagnosed with cardiomyopathy and developed an intracardiac thrombus or distant thromboembolic event.

Exclusion criteria:

- Patients with congenital heart disease that required a mechanical valve insertion.
- Patients with dilated cardiomyopathy secondary to Fontan procedure and arrhythmogenic right ventricular cardiomyopathy.

From January 2017 to April 2019, out of 180 pediatric patients who are known to have cardiomyopathy, nine cases had the complications of interest for which data regarding age, gender, date of diagnosis, the onset of complications, cardiac hemodynamics and details of each complication were collected.

This study project was conducted following the ethical principles contained in the Declaration of Helsinki (2000), the ICH Harmonized Tripartite Good Clinical Practice Guidelines, the policies and guidelines of the Research Advisory Council of the KFSHRC, and the laws of Saudi Arabia.

## 3 RESULTS

Out of 180 patients (0‐14 years old) following‐up at the cardiomyopathy clinic in KFSHRC. We have identified nine cases presenting with intracardiac thrombi or cardiac‐related thromboembolic events. Five male and four female patients. Seven out of the nine patients were diagnosed with dilated cardiomyopathy whereas the remaining two were diagnosed with restrictive type.

The mean age of onset was 5.6 years. Ross classification for heart failure in children was used to identify and score the status of the clinical presentation of all patients, which showed six patients have presented with symptoms of heart failure at rest, two patients had the symptoms with mild exercise and only one patient experienced symptoms with moderate exercise.^8^

Left ventricular ejection fraction (LVEF) was ≤ 25% in all patients with dilated cardiomyopathy. On the other hand, the two patients who were diagnosed with restrictive cardiomyopathy had an LVEF of 26% and 40% at the time of presentation. Further details about cardiac hemodynamics are shown in (Table 1).

**Table 1:** Age, gender and cardiac hemodynamics (MR, LA dilation, LVEDD and LVESD z‐scores)

Patients	Age (Year) at onset of thrombus/embolus	Gender	LVEF	FS	MR	LAD	LVEDD z‐score	LVESD z‐score
Case 1	12	Male	24%	17.3%	Moderate	Severe	3	6.7
Case 2	10	Male	12%	12.4%	Mild	Moderate	15	18.5
Case 3	3	Male	25%	9.5%	Trivial	Moderate	2.3	7.2
Case 4	5	Female	14%	6.4%	Mild	Moderate	8.7	15
Case 5	1	Male	19%	13.5%	Mild	Severe	9.1	13.8
Case 6	1	Female	20%	14.4%	Mild	Severe	4.3	8.4
Case 7	11	Female	>40%	28.6%	Mild	Severe	‐2.9	‐1.2
Case 8	7	Female	26%	18%	Mild	Severe	0.3	2.7
Case 9	1	Male	25%	13.7%	Moderate	Mild	8.3	12.8

Note

Abbreviations: FS, Fractional shortening; LAD, Left atrial dilatation; LVEDD, Left ventricular end‐diastolic diameter; LVEF, Left ventricular ejection fraction; LVESD, Left ventricular end‐diastolic diameter; MR,: Mitral regurgitation.

Reviewing the use of anticoagulant/antiplatelet medications revealed that 6 out of 9 patients were started on aspirin (81 mg, daily) before their presentation out of which 50% presented with thromboembolic events. Aspirin was the only type of anticoagulant/antiplatelet that was used in our studied population. None of the patients is known to have any hematological or thrombotic diseases and there was no family history of such diseases either.

One patient of the dilated cardiomyopathy group had a significant family history of dilated cardiomyopathy in a first‐degree cousin for which whole‐exome sequencing (WES) was sent and came back positive for (PPP1R13L) gene mutation.

After clear identification and management, the outcome was complete resolution of the intracardiac thrombus in three patients evident by follow‐up echocardiography, partial recovery of thrombus‐related neurological complications in two patients, permanent neurological insults in form of motor and sensory dysfunctions in three patients and unfortunately, death was the outcome of one patient. Description of the intracardiac thrombosis along with other thromboembolic events and circumstances of each patient are listed in (Table 2).

**Table 2:** Circumstances of intracardiac thrombosis and systemic embolism

Patients	Type of anticoagulant/antiplatelet prior to the onset of thrombosis or embolism	Site and shape of intracardiac thrombus	Site of systemic embolus	Outcome
Case 1	Aspirin	Apex Mural/ immobile	No embolization	No embolization
Case 2	Aspirin	NOT AVAILABLE	Basal ganglia and right MCA	Permanent neurological insult
Case 3	None	NOT AVAILABLE	Left and right MCA	Death
Case 4	Aspirin	Anterior wall Intracavitary/ small mobile extension	No embolization	No embolization
Case 5	Aspirin	Anterior wall Mural/ immobile protuberant	No embolization	No embolization
Case 6	Aspirin	NOT AVAILABLE	Left MCA	Permanent neurological insult
Case 7	None	NOT AVAILABLE	Left iliac artery	Partial recovery of motor dysfunction
Case 8	None	NOT AVAILABLE	Left MCA	Permanent neurological insult
Case 9	Aspirin	NOT AVAILABLE	Left MCA	Partial recovery of motor dysfunction

Note

Abbreviation: MCA, Middle cerebral artery.

**Figure 1:**
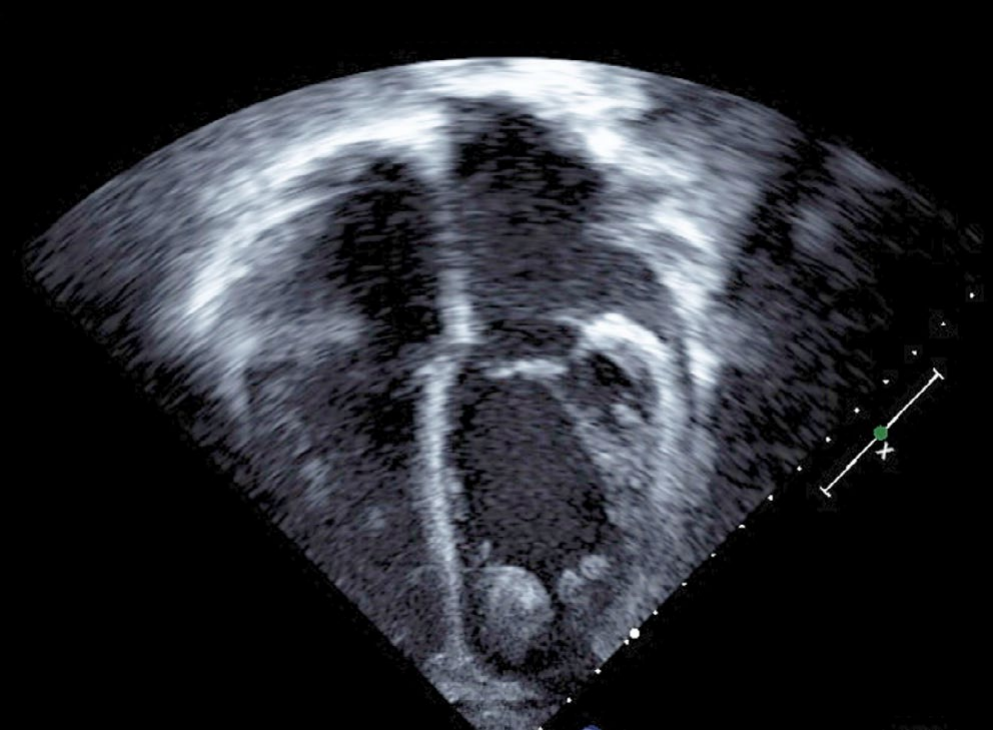
Echocardiography of Case 1 showing Multiple clots seen in left ventricular apex

**Figure 2:**
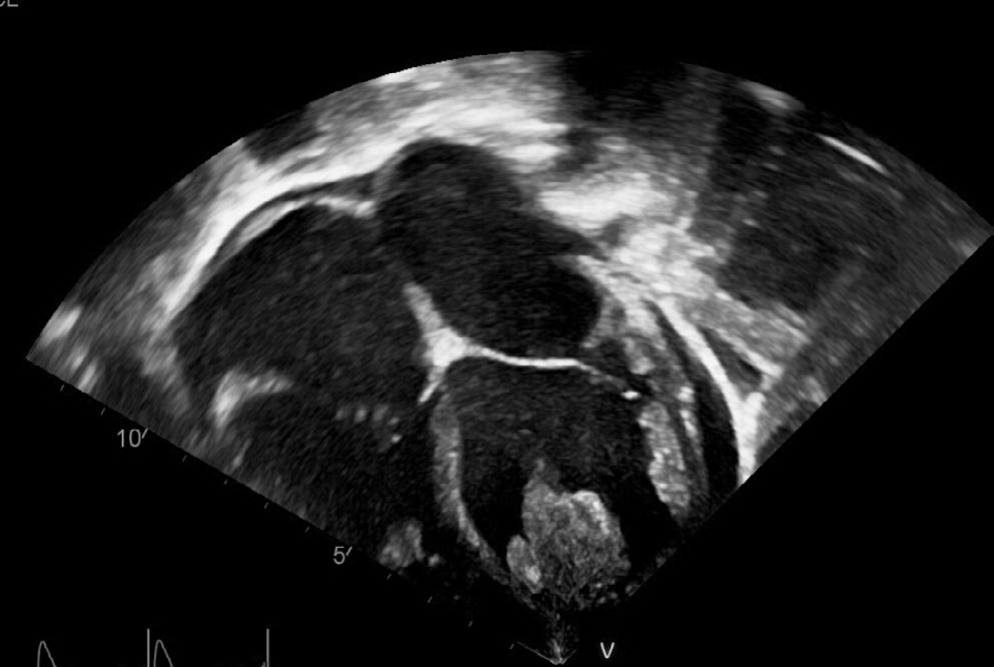
Echocardiography of Case 4 showing Large thrombus seen in the left ventricle, attached to the anterior septum and anterior wall (wide base) with multiple lobes and some mobile extensions

**Figure 3:**
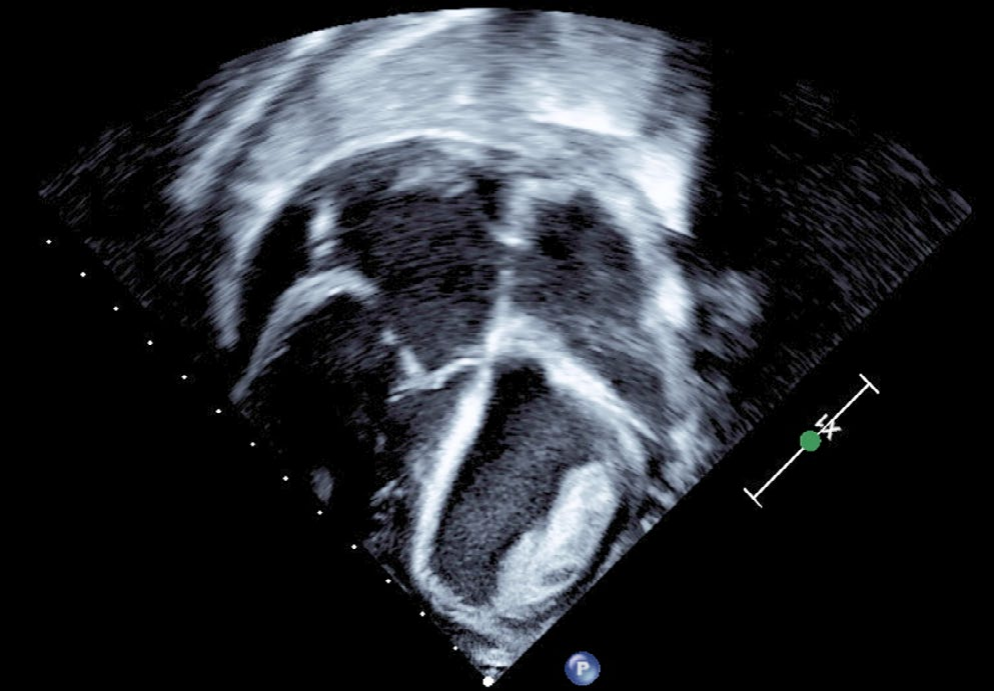
Echocardiography of Case 5 showing large echogenic mass attached to the anterior left ventricular wall

Successful detection of intracardiac thrombosis was achieved upon presentation of cases 1, 4, and 5 who presented with symptoms of heart failure (Figures 1, 2, 3). They were promptly treated as shown in (Table 3) without any neurological sequelae.

**Table 3:** Diagnosis and management of thrombosis or thromboembolic events

Patients	Diagnosis	Management
Case 1	Dilated cardiomyopathy	Warfarin followed by subcutaneous enoxaparin
Case 2	Dilated cardiomyopathy	Heparin infusion followed by subcutaneous heparin
Case 3	Dilated cardiomyopathy	Subcutaneous enoxaparin
Case 4	Dilated cardiomyopathy	Heparin infusion followed by subcutaneous heparin
Case 5	Dilated cardiomyopathy	Subcutaneous heparin
Case 6	Dilated cardiomyopathy	Referred with complications “unknown treatment”
Case 7	Restrictive cardiomyopathy	Heparin infusion followed by warfarin
Case 8	Restrictive cardiomyopathy	Heparin infusion followed by warfarin
Case 9	Dilated cardiomyopathy	Referred with complications “unknown treatment”

Note

Doses of each pharmacological agent were chosen following an evidence‐based drug formulary depending on the patient age and weight with the aid of the clinical pharmacists.

## 4 DISCUSSION

Cardiomyopathy is a leading cause of heart failure and it is considered as one of the most contributing causes in cardiac complications and heart transplantation in the pediatric population. A variety of complications and health issues can occur in patients with cardiomyopathies among which comes cardiac‐related thrombus formation and thromboembolic events. Although rare, this phenomenon is fatal and can significantly alter the prognosis of the patient due to the huge effect of the disease process on the patient's general health status and all the subsequent interventions that would normally follow including pharmacological agents, imaging, and medical procedures. Patients are usually admitted to a critical care unit to be followed closely and this admission carries its risks as well.

Two‐dimension echocardiography is the most commonly used modality to follow‐up patients with cardiomyopathies and to diagnose intracardiac thrombosis if suspected. However, with a sensitivity of (92%) and a specificity of (88%) the possibility of having undetectable intracardiac thrombus still stands.^5^

Aspirin which is an antiplatelet agent is used as an anticoagulant in these cases. In a multicenter, double‐blinded, randomized trial by J.P Mohr et al who compared the effect of warfarin and that of aspirin for the prevention of recurrent ischemic stroke has found that both agents are reasonable therapeutic alternatives without any significant difference.^9^

Chen K et al have published a review to examine the incidence and risk factors associated with embolism in the pediatric population with dilated cardiomyopathy (DCM), restrictive cardiomyopathy (RCM), and noncompaction of the left ventricular myocardium (NLVM). The review concluded by stating the difficulty of establishing a unified algorithm for embolic management due to the ongoing challenges in decision making regarding the types of anticoagulants, the optimal duration of therapy, efficacy, and the fact that the initiation of anticoagulation can promote embolism in certain cases.^10^

Despite the great effort that has been made by some centers and physicians who have reported similar complications, it appears that there is still no clear consensus on how to prevent, closely follow or manage such unfortunate events. This is not surprising giving the fact that the predisposing condition is considered multifactorial and practically lacks clear etiological and prognostic factors.^11^

It was noted from our results and that of other reports the age discrepancy at the time of diagnosis, the duration from symptoms to diagnosis, and from diagnosis to complications. Combining the previously mentioned points, it is important to consider the missing factors that could either prevent these complications or improve their outcomes and of how many undetected or unreported cases are there all over the world in order to establish global diagnostic and therapeutic protocols with such patients.

## 5 CONCLUSION

Intracardiac thrombosis or distant thromboembolic events are rare complications in patients diagnosed with any type of cardiomyopathies. The low prevalence of these complications makes it challenging for cardiovascular practice and unfortunate to the patients and their families. We believe that more reports are needed to extensively study such complications and to encourage specialized health care providers to present lifesaving measures in the near future.

## CONFLICT OF INTEREST

The authors declare that they have no conflict of interest.

## AUTHOR CONTRIBUTION

Abdulrahman Bendahmash AB, Saeed Almanie SA, and Abdullah Alwadai AA. AB and SA: assisting with identifying appropriate patients, reviewing medical records, data collection and analysis, writing the manuscript. AA: overall conduction of the study including reporting to the Research Advisory Council, identifying appropriate patients, reviewing medical records, data collection and analysis, and writing the manuscript.
